# Early maxillary arch development in unilateral cleft lip and palate: a retrospective 3D morphometric analysis following nasoalveolar molding and cheiloplasty

**DOI:** 10.1186/s12903-026-07754-4

**Published:** 2026-01-28

**Authors:** Thanaporn Pattanawasin, Jutharat Chimruang, Saran Worasakwutiphong, Ratchawan Tansalarak

**Affiliations:** 1 Department of Preventive Dentistry, Faculty of Dentistry, Naresuan University, Phitsanulok, 65000 Thailand; 2 Department of Surgery, Faculty of Medicine, Naresuan University, Phitsanulok, 65000 Thailand; 3 Naresuan University Cleft and Craniofacial Center, Phitsanulok, 65000 Thailand

**Keywords:** Cleft lip, Cleft palate, Nasoalveolar molding, Cheiloplasty, Three-dimensional image, Dental arch

## Abstract

**Background:**

Early assessment of maxillary arch development is crucial in infants with unilateral cleft lip and palate (UCLP). This study aimed to evaluate three-dimensional changes in the maxillary arch following modified nasoalveolar molding (NAM) and cheiloplasty using digital model analysis.

**Methods:**

This retrospective study included 25 infants with complete UCLP who underwent modified NAM therapy prior to cheiloplasty. Digital maxillary models were obtained at two time points: T1 on the day of cheiloplasty at 3–6 months of age and T2 on the day of palate repair at 9–18 months of age. Three-dimensional linear and angular measurements were performed to assess transverse, sagittal, and vertical dimensional changes. Changes between T1 and T2 were analyzed using paired t-tests or Wilcoxon signed-rank tests, depending on data distribution, with a significance level set at *p* < 0.05. Digital model superimposition based on posterior maxillary reference landmarks was used to visualize morphological changes over time.

**Results:**

Significant changes were observed in both the transverse and sagittal dimensions. The anterior cleft width decreased significantly (mean difference = − 0.95 mm, *p* = 0.001), whereas the anterior and posterior arch widths increased (mean difference = 1.81 mm and 0.99 mm, respectively). The lengths of both the greater and lesser segments increased significantly, and a medial rotation of the greater segment was noted. However, no significant changes were observed in the vertical dimension. Superimposition of the digital models demonstrated notable medial movement of the greater segment and narrowing of the alveolar cleft gap.

**Conclusions:**

This study provides preliminary data on early morphological changes in the maxillary arch following modified NAM and cheiloplasty in infants with UCLP. Although no control group was included, three-dimensional assessment revealed favorable growth patterns in the transverse and sagittal dimensions. Further prospective studies with control comparisons are recommended to confirm these findings.

## Background

Cleft lip and palate (CLP) are among the most common congenital craniofacial anomalies, with a reported prevalence of approximately 2.14 per 1000 live births in Thailand [1]. The etiology of CLP involves both congenital dysmorphogenesis and postnatal growth disturbances, which are often exacerbated by scarring from surgical repair. Patients with unrepaired CLP typically exhibit near-normal midfacial and dental arch development, whereas those undergoing surgical interventions may experience midfacial retrusion and maxillary growth restriction [2–7].

Cheiloplasty, typically performed at 3–6 months of age, is a crucial procedure in CLP management that aims to restore lip form, function, and aesthetics by approximating the cleft segments. However, this reconstruction may induce soft tissue tension and scar formation, which have been suggested to influence maxillary morphology. Experimental and clinical studies indicate that increased lip pressure may be associated with altered craniofacial development [8–10], and clinically, post-cheiloplasty approximation of the alveolar segments and changes in anterior maxillary width have been reported in most infants [11]. The early post-cheiloplasty period therefore represents a phase of active tissue adaptation and early maxillary growth, during which morphological changes may be clinically relevant for planning subsequent interventions, such as palatoplasty or adjunctive orthopedic management [6, 12, 13]. Despite increasing use of three-dimensional (3D) imaging techniques, objective 3D data describing maxillary arch changes across all spatial dimensions during the early post-cheiloplasty period remain limited [14].

Presurgical nasoalveolar molding (NAM) is widely used to align alveolar segments and improve nasal symmetry prior to cheiloplasty. At the Naresuan University Cleft and Craniofacial Center, a modified NAM protocol based on the technique from Chang Gung Memorial Hospital is routinely utilized [15, 16]. This appliance reduces the alveolar gap and reshapes nasal cartilage, facilitating improved surgical outcomes [17, 18]. While many studies have focused on the nasolabial outcomes of NAM, relatively few have evaluated changes in maxillary arch morphology, and even fewer have assessed these changes following cheiloplasty.

3D digital models allow accurate, reproducible, and efficient evaluation of dental arch morphology, with advantages over traditional plaster models such as long-term data storage and ease of data sharing [19–23].

Therefore, this retrospective study aimed to evaluate early maxillary arch development in infants with unilateral cleft lip and palate (UCLP) following modified NAM and cheiloplasty, using 3D morphometric analysis to quantify changes in the transverse, sagittal, and vertical planes.

## Methods

### Study design and ethical approval

This retrospective study was approved by the Naresuan University Institutional Review Board (IRB No. P1-0120/2567). It was based on the work by Chaisooktaksin et al. [16], which demonstrated the effectiveness of a modified NAM device in reducing alveolar cleft width and aligning maxillary segments prior to cheiloplasty in infants with complete UCLP. The current study aimed to assess subsequent 3D maxillary arch changes after cheiloplasty using digital model analysis.

The sample size was calculated using G*Power version 3.1 (Heinrich-Heine-Universität, Düsseldorf, Germany) on the basis of an effect size of 0.64 derived from a previous study by Pai et al. [24] to achieve a power of 0.80 at a significance level of 0.05, and a minimum of 22 patients was needed. To account for a 10% dropout rate, 25 subjects were recruited.

### Subjects

This study included 25 infants who were diagnosed with non-syndromic UCLP and who underwent modified NAM therapy followed by cheiloplasty between March 2014 and June 2024. All patients met the inclusion criteria of having non-syndromic complete UCLP and receiving NAM treatment by a single orthodontist at the Faculty of Dentistry, Naresuan University.

All surgical procedures were performed by a single plastic surgeon at the Faculty of Medicine, Naresuan University, ensuring consistency in both orthodontic and surgical protocols.

Complete medical records were available for all included subjects, and relevant data, including demographic information, NAM treatment details, surgical procedures, and postoperative outcomes, were systematically collected using predetermined data extraction forms.

### Modified NAM procedure

The modified NAM device was adapted from the Chang Gung Memorial Hospital protocol and used routinely at the Naresuan University Cleft and Craniofacial Center [15, 16]. It consisted of a hard acrylic molding plate with a 0.036-inch stainless-steel nasal stent, which was retained using extraoral taping.

**Fig. 1:**
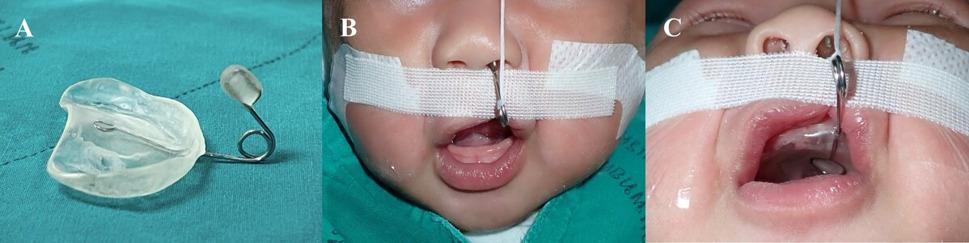
(**A**) Modified NAM appliance (**B**) Frontal view of an infant with complete UCLP undergoing modified NAM therapy (**C**) Basal view of the same patient showing the intraoral molding plate and nasal stent

At the first visit, clinical examinations were conducted, and detailed treatment information was provided to the caregivers. Upon providing informed consent and confirming the infant’s health, modified NAM therapy commenced (Fig. 1). Adjustments, including overcorrection of the cleft nostril and adequate columellar elongation, were scheduled every 2–4 weeks until the desired nasal and alveolar molding was achieved. A maxillary impression was obtained on the day of cheiloplasty for the T1 model.

### Surgical procedure

Cheiloplasty and primary rhinoplasty were performed by a single surgeon via a modified rotation-advancement technique based on Noordhoff’s method, along with Tajima’s technique for nasal correction. Lip repair was performed when the infants were between 3 and 6 months of age. A second impression was taken on the day of palate repair, prior to the surgical procedure, when the infants were between 9 and 18 months old, to obtain the T2 model.

### Data collection and 3D model analysis

Maxillary models were scanned via an iTero Element 2 intraoral scanner (Align Technologies; San Jose, CA). The digital files (.stl) were analyzed using Blender software version 4.0 (Blender Foundation; Amsterdam, the Netherlands).

**Fig. 2:**
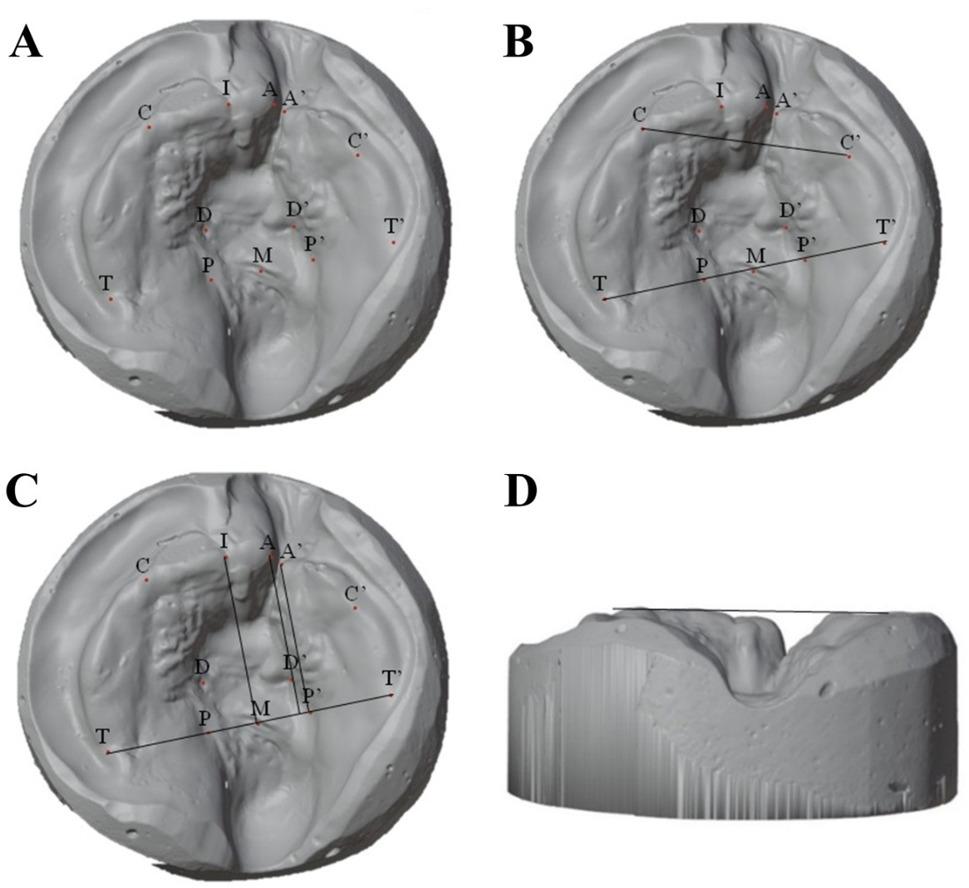
(**A**) Positioning of reference points for the measurement (**B**) Transverse measurements (**C**) Sagittal and angular measurements 1, Anterior cleft width (A-A’); 2, Posterior cleft width (P-P’); 3, Anterior arch width (C-C’); 4, Posterior arch width (T-T’); 5, Length of the greater cleft segment (A⊥T-T’); 6, Length of the lesser cleft segment (A’⊥T-T’); 7, Greater segment rotation (IMT angle) (**D**) Horizontal reference plane

Reference points were defined according to a previous study [14] and marked using Blender’s Annotate tool (Fig. 2; Table 1). Linear and angular measurements were obtained using the Measure tool. The T1 and T2 models were superimposed on the maxillary tuberosity points, which were selected as reference landmarks for superimposition. The maxillary tuberosity region was chosen because it is considered relatively stable during early infancy and is less directly affected by cheiloplasty-related surgical manipulation at the cleft site. This approach has been applied in previous studies to minimize the influence of localized surgical remodeling on superimposition accuracy [25].

**Table 1:** Definitions of reference points, reference planes, and measurements

Reference points	Definition
Incisal point (I)	The intersection of the crest of the alveolar ridge and the line drawn from the labial frenum to the incisive papilla
Anterior cleft point (GS-A) (LS-A’)	The anterior endpoint of the alveolar crest (at the most inferior, medial, midpoint of the alveolar crest). Where A is the anterior cleft point on the greater segment and A’ is the anterior cleft point of the lesser segment.
Canine points (GS-C) (LS-C’)	The intersection of the lateral sulcus and the crest of the alveolar ridge
Tuberosity points (GS-T) (LS-T’)	The posterior endpoint of the alveolar crest (intersection of the crest of the alveolar ridge with the outline of the tuberosity)
Posterior cleft points (GS-P) (LS-P’)	The intersection of the alveolar cleft margin with the plane that connects the tuberosity points
Deep cleft (GS-D) (LS-D’)	The deepest point of the cleft segment
M point	The midpoint of the line connecting the tuberosity point
Horizontal plane of the greater segment (HP)	The horizontal plane passing through the C point
Measurements	
A-A’	Anterior cleft width
P-P’	Posterior cleft width
C-C’	Anterior arch width
T-T’	Posterior arch width
A⊥T-T’	Length of the greater cleft segment
A’⊥T-T’	Length of the lesser cleft segment
D-HP	Height of the greater cleft segment (distance from the deepest point of the greater cleft segment perpendicular to the horizontal plane of the greater segment)
D’-HP’	Height of the lesser cleft segment (distance from the deepest point of the lesser cleft segment perpendicular to the horizontal plane of the lesser segment)
IMT angle	Greater segment rotation

Nevertheless, potential methodological limitations should be considered, such as growth-related changes in posterior maxillary structures, variability in landmark identification, and the absence of a true cranial reference system, which may influence the precision of vertical and angular measurements [25, 26].

### Statistical analysis

Descriptive statistics were used to summarize the data. Paired t- tests were applied to compare normally distributed variables between T1 and T2. For non-normally distributed variables, the Wilcoxon signed-rank test was used. A *p*-value less than 0.05 was considered statistically significant.

To assess intraexaminer reliability, 5 randomly selected models were re-measured by the same examiner after a two-week interval. The intraclass correlation coefficients (ICCs) ranged from 0.976 to 1.000, indicating excellent reproducibility [27].

## Results

A total of 25 infants with non-syndromic complete UCLP were included in this study, comprising 17 males (68%) and 8 females (32%). The mean birth weight was 3000.8 ± 295.7 g. Clefts were located on the right side in 7 patients (28%) and on the left side in 18 patients (72%). The mean age at the first NAM appointment was 22.04 ± 23.31 days. The mean age at the time of cheiloplasty (T1) was 120.72 ± 26.05 days, and that at the time of palatoplasty (T2) was 350.52 ± 55.93 days. The mean initial alveolar cleft width before treatment was 9.84 ± 3.66 mm. Patient demographic data are summarized in Table 2.

**Table 2:** Demographic and clinical characteristics of infants with complete unilateral cleft lip and palate (*N* = 25)

Characteristic	*n* (%)	Mean ± SD	Range
Gender			
Male	17 (68)	-	-
Female	8 (32)	-	-
Cleft Side			
Left	18 (72)	-	-
Right	7 (28)	-	-
Birth weight (g)	25 (100)	3000.8 ± 295.7	2290–3420
Initial cleft width (mm)	25 (100)	9.84 ± 3.66	2.36–15.71
Age at treatment milestones (days)			
First NAM appointment	25 (100)	22.04 ± 23.31	4–98
Cheiloplasty (T1)	25 (100)	120.72 ± 26.05	89–173
Post-cheiloplasty (T2)	25 (100)	350.52 ± 55.93	310–527

### Maxillary arch changes between T1 and T2

Quantitative analysis of the 3D digital models revealed statistically significant dimensional changes between T1 and T2 (Table 3).

**Table 3:** Changes in maxillary arch dimensions between T1 and T2 in infants with unilateral cleft lip and palate (N = 25)

Parameter (mm)	Cheiloplasty (T1) Mean ± SD	Post- Cheiloplasty (T2) Mean ± SD	Mean Difference (T2-T1) Mean ± SD	95% CI	*P*-Value
Cleft width					
Anterior (A-A’)	3.84 ± 2.49	2.89 ± 2.52	-0.95 ± 1.28	-1.48, -0.42	**0.001a**
Posterior (P-P’)	11.81 ± 2.95	12.06 ± 4.06	0.25 ± 2.16	-0.64, 1.14	0.567b
Arch width					
Anterior (C-C’)	27.43 ± 3.39	29.24 ± 3.70	1.81 ± 2.94	0.60, 3.03	**0.005b**
Posterior (T-T’)	33.55 ± 2.51	34.54 ± 2.30	0.99 ± 2.04	0.15, 1.84	**0.023b**
Cleft segment dimensions					
Greater segment length	21.71 ± 2.69	22.80 ± 2.20	1.09 ± 2.55	0.04, 2.15	**0.043b**
Lesser segment length	19.13 ± 2.15	20.89 ± 2.07	1.76 ± 2.43	0.76, 2.77	**0.001b**
Greater segment height (D-HP)	8.61 ± 1.64	9.07 ± 1.68	0.46 ± 2.18	-0.44, 1.36	0.288a
Lesser segment height (D’-HP’)	8.48 ± 1.58	8.65 ± 1.68	0.17 ± 1.83	-0.59, 0.92	0.649b
Greater segment rotation (IMT)	82.12° ± 5.45°	84.04° ± 6.11°	1.92° ± 4.17°	0.20, 3.64	**0.030b**

All measurements are in millimeters except for the IMT angle (degrees)

T1 = at cheiloplasty; T2 = at post-cheiloplasty (mean 229.8 days after T1)

Boldface indicates statistically significant values *P* < 0.05

**a** Wilcoxon signed-rank test

**b** Paired t-tests

In the transverse dimension, the anterior cleft width decreased significantly by 0.95 ± 1.28 mm (*p* = 0.001). The posterior cleft width showed a small increase of 0.25 ± 2.16 mm, which was not statistically significant (*p* = 0.567). Both anterior and posterior arch widths demonstrated significant increases. The anterior arch width increased by 1.81 ± 2.94 mm (*p* = 0.005), while the posterior arch width increased by 0.99 ± 2.04 mm (*p* = 0.023).

In the sagittal dimension, significant increases in segment length were observed in both the greater and lesser segments. The length of the greater segment increased by 1.09 mm (*p* = 0.043), and the length of the lesser segment increased by 1.76 mm (*p* = 0.010).

In the vertical dimension, changes in vertical height were minimal and did not reach statistical significance. The vertical height of the greater segment increased by 0.46 ± 2.18 mm (*p* = 0.288), while that of the lesser segment increased by 0.17 ± 1.83 mm (*p* = 0.649).

Additionally, a statistically significant increase in the IMT angle was observed, with a mean change of 1.92° ± 4.17° (*p* = 0.030), indicating a medial rotational change of the greater segment.

### Digital superimposition analysis

**Fig. 3:**
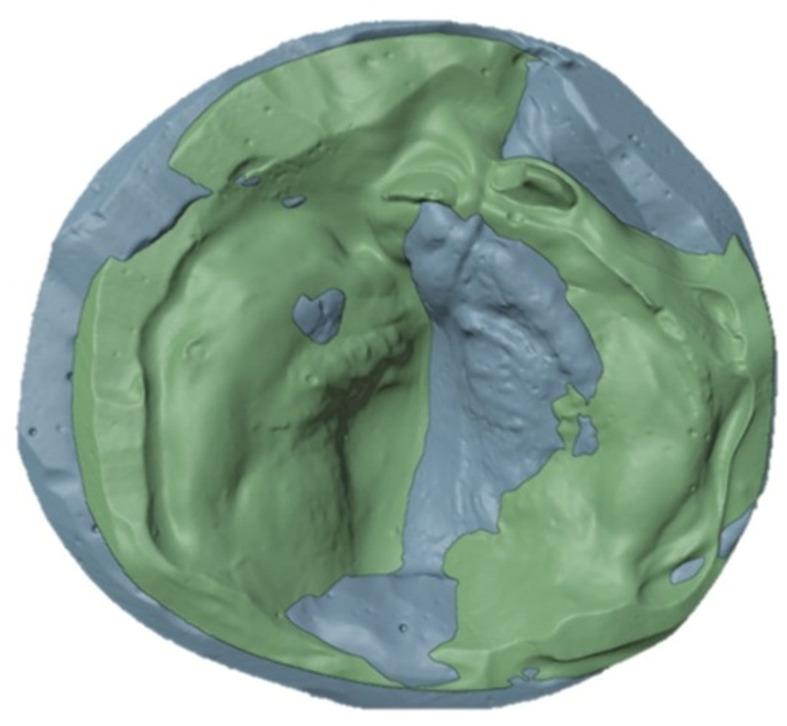
Superimposition of maxillary models at T1 and T2 using posterior maxillary reference landmarks (blue, T1; green, T2)

3D superimposition of maxillary models obtained at T1 (blue) and T2 (green), using posterior maxillary reference landmarks, demonstrated medial displacement and rotational changes of the anterior portion of the greater segment (Fig. 3). In addition, increases in both anterior and posterior arch widths were observed, consistent with transverse dimensional changes identified in the quantitative analysis. The superimposition further showed medial movement of the anterior segment associated with a reduction in the alveolar cleft width.

## Discussion

The management of CLP aims to restore function, facial aesthetics, and harmonious craniofacial growth. Advances in surgical techniques and the integration of presurgical NAM have contributed to improved early outcomes in patients with CLP [28–30]. Cheiloplasty, commonly performed between 3 and 6 months of age [31], plays a central role in reconstructing the lip and nasal structures (16), however, its influence on subsequent maxillary growth remains controversial. Excessive scarring following lip and palatal repair has been associated with maxillary arch constriction [32], midfacial retrusion [33], and posterior crossbites [32]. Therefore, understanding early post-cheiloplasty changes in maxillary morphology is essential for optimizing treatment protocols and improving long-term craniofacial outcomes.

Different treatment protocols are used across centers [34], and several comparative studies have examined the effects of various presurgical orthopedic approaches. Both passive alveolar molding (PAM) and NAM have been shown to reduce anterior cleft width, with some studies reporting greater reductions with NAM compared with PAM [35]. In addition, simpler approaches such as lip taping have been proposed as cost-effective alternatives and may be particularly useful in low-resource settings [36].

Building on the work of Chaisooktaksin et al. [16], the present study extends the evaluation of maxillary morphology beyond the pre-cheiloplasty period by analyzing changes between cheiloplasty and palatoplasty using 3D digital model. Compared with conventional plaster models, digital 3D analysis provides improved reproducibility, accuracy, and efficiency for longitudinal assessment [19–23].

The present findings demonstrated significant reductions in anterior cleft width and significant increases in both anterior and posterior arch widths during the early post-cheiloplasty period. Sagittal elongation of both maxillary segments was also observed. These results are consistent with previous studies reporting transverse expansion and anterior maxillary growth following NAM and lip repair [32, 37]. In contrast, vertical dimensions remained relatively stable, which aligns with earlier reports indicating limited vertical maxillary change during infancy [16, 33]. Overall, these findings suggest that morphological changes during the early post-cheiloplasty period tend to be more pronounced in the transverse and sagittal dimensions.

A significant increase in the IMT angle indicated medial rotational change of the greater segment. This finding may reflect the combined influence of the modified NAM and the soft tissue forces introduced by cheiloplasty, as suggested by experimental and clinical studies describing the effects of lip pressure and scarring on maxillary morphology [8–10, 33]. Such rotational changes may contribute to improved approximation and alignment of the alveolar segments during early development [35]. While modified NAM likely plays a major role in preoperative segment alignment, the post-cheiloplasty changes observed in this study may represent a synergistic influence of presurgical orthopedic treatment and surgical intervention on maxillary morphology [32]. Although the absence of a control group limits causal inference, the consistent trends toward improvement across transverse, sagittal, and rotational dimensions help contextualize early morphological adaptation during the post-cheiloplasty period.

From a clinical perspective, the observed transverse widening, sagittal elongation, and segmental rotation may contribute to a more coordinated maxillary arch form prior to palatoplasty [38]. Improved alignment of the alveolar segments could potentially facilitate surgical closure and reduce tissue tension during subsequent procedures [39]. Moreover, early normalization of arch morphology may help simplify later orthodontic management, although this hypothesis requires confirmation through long-term follow-up studies [32].

Despite these findings, several limitations must be acknowledged. The retrospective design may introduce selection and measurement bias. The absence of a non-treated or non-cleft control group limits causal inference regarding the effects of NAM and cheiloplasty on maxillary growth. In addition, individual variability in cleft severity and segmental hypoplasia, particularly of the lesser segment, may influence observed outcomes [40]. Finally, the relatively short observation period precludes conclusions regarding long-term craniofacial growth patterns.

## Conclusions

This study provides a three-dimensional evaluation of early maxillary arch development in infants with non-syndromic complete UCLP treated with modified NAM followed by cheiloplasty. Significant changes were observed in the transverse and sagittal dimensions between cheiloplasty and palatoplasty, including reductions in anterior cleft width, increases in anterior and posterior arch widths, elongation of both maxillary segments, and medial rotation of the greater segment. In contrast, vertical dimensions remained relatively stable during this period.

These findings suggest that the combination of modified NAM and standardized cheiloplasty is associated with coordinated early changes in maxillary morphology across multiple spatial dimensions. However, in the absence of a control group and long-term follow-up, it cannot be determined whether these changes reflect normal growth patterns or are attributable to treatment-related influences. Future prospective studies incorporating appropriate control groups and extended observation periods are needed to clarify the long-term effects of early cleft interventions on maxillofacial development.

## Data Availability

The datasets used and/or analyzed during the current study are available from the corresponding author upon reasonable request.

## Abbreviations

CLP: Cleft lip and palate
3D: Three-dimensional
NAM: Nasoalveolar molding
UCLP: Unilateral cleft lip and palate
PAM: Passive alveolar molding
